# Combining UPLC/Q-TOF-MS/MS With Biological Evaluation for NF-κB Inhibitors in Uyghur Medicine *Althaea rosea* Flowers

**DOI:** 10.3389/fpls.2018.01975

**Published:** 2019-01-09

**Authors:** Fang Ma, Qingxin Cui, Gang Bai

**Affiliations:** Tianjin Key Laboratory of Molecular Drug Research, College of Pharmacy and State Key Laboratory of Medicinal Chemical Biology, Nankai University, Tianjin, China

**Keywords:** constituent identification, NF-κB inhibitors, UPLC/Q-TOF-MS/MS, *Althaea rosea* (Linn.) flowers, anti-inflammatory compounds

## Abstract

The *Althaea rosea* (Linn.) flower is a common plant that is often used to control inflammation in Uyghur ethnic medicine. However, its active ingredients remain uncertain and difficult to identify, severely limiting its use as a valuable crop. This paper aims to establish a rapid assay strategy for the integration of ultra-performance liquid chromatography/quadrupole time-of-flight mass spectrometry (UPLC/Q-TOF-MS/MS) and a biologically active (NF-κB inhibitor) luciferase reporter detection system to explore various anti-inflammatory compounds of *A. rosea* (Linn.) flowers. Potential anti-inflammatory components were screened using the NF-κB activity assay system and simultaneously identified based on mass spectrometry data. Four structural types of NF-κB inhibitors (phenolic acid, hydroxycinnamic acid, flavonoid, and dihydroflavone) were identified. Further cytokine assays confirmed their potential anti-inflammatory effects as NF-κB inhibitors. Compared with traditional chromatographic separation, integrated UPLC/Q-TOF-MS/MS identification compounds, and biological activity verification are more convenient and more reliable. This strategy clearly demonstrates that fingerprinting based on MS data not only can identify unknown components but also is a powerful and useful tool for screening trace active ingredients directly from complex matrices. *A. rosea* (Linn.) exhibits great health and pharmaceutical value and may contribute to the development of new anti-inflammatory drugs.

## Introduction

*Althaea rosea* (Linn.) (Althaea, Malvaceae) is a common perennial ornamental plant (Zhang et al., 2015), generally known as hollyhock or marshmallow, and is usually grown in gardens, parks, river banks, and salt marshes. The plant is native to China and is now found in tropical and temperate regions around the world, including the Middle East, the Mediterranean, Central Asia, and Southern Europe (Choi et al., 2012). The medicinal parts of *A. rosea* (*A. rosea*) include flowers, leaves, roots, and seeds (McKeown et al., 2013). *A. rosea* flowers have been used in traditional Uyghur medicine to treat a variety of diseases for a long time as anti-inflammatory agents, febrifuge, palliatives, and astringents (Kwiatkowska et al., 2011). However, the material basis of its anti-inflammatory effects remains to be elucidated (Normile, 2003). Therefore, the isolation and identification of small molecules and their biological activities are important for understanding the mode of action (MOA) of *A. rosea* flowers and their effects on physiology (Zhang et al., 2008). Under these conditions, ultra-performance liquid chromatography coupled with time-of-flight mass spectrometry (UPLC/Q-TOF-MS/MS) provides accurate structural information about the bioactive compounds for the separation and identification of mixtures (Xu et al., 2017; Ye et al., 2017). High throughput screening based on biological active systems is a rapid method of assaying potential inhibitors against a specific target (Klimes et al., 2017; Aburai et al., 2018). The combination of the two methods can quickly provide structural and activity information for complex samples and provides a basis for the screening of pharmacological substances.

Inflammation is a basic pathological process in which a biological tissue is stimulated by trauma or infection to promote a defensive response. The main consequence of the increase in inflammatory signaling is the upregulation of nuclear factor-κB (NF-κB) and subsequent damage, and the intensity of the damage depends on the type of activation of the NF-κB dimer (Kim et al., 2013; Srinivasan and Lahiri, 2015). NF-κB plays a key role in the expression of many pro-inflammatory genes caused by viral and bacterial infections. This expression leads to the synthesis of cytokines and chemokines, including interleukins IL-6, IL-8, RANTES, IL-11 and eosinophil chemotactic factors (Edwards et al., 2009; Legan et al., 2015; Zyuz’kov et al., 2015), causing an inflammatory stress response. Screening based on NF-κB inhibitory activity will help to identify effective and novel anti-inflammatory drugs (Cheng et al., 2012).

The inflammatory effect is achieved through activation of phagocytic activity, increased expression of NF-κB and chemokines (including tumor necrosis factor (TNF-α, IL-1, IL-6, IL-8, and IL-12) (Stockley et al., 2017). Many reports have demonstrated that LPS (lipopolysaccharide) treatment can stimulate cells to increase NO, ROS, and cytokine production (Ando et al., 2002). Selecting appropriate cell lines for *in vitro* models is a useful method to evaluate immunomodulatory effects by measuring the synthesis of inflammatory molecules in response to different stimuli. This provides an effective clue for screening the core structure of natural products and developing effective small molecule inhibitors that selectively target NF-κB activation.

In this paper, an integrated strategy combining UPLC/Q-TOF-MS/MS with biological evaluation for NF-κB inhibitors was proposed. *A. rosea* flowers were investigated using the combined method of chemical component identification and bioactivity detection. Potential bioactive compounds were identified according to the mass spectrometry data and screened by NF-κB activity assay system simultaneously. In conjunction with our subsequent studies of enzyme-linked immune sorbent assay (ELISA) evaluation of inflammatory factors, the anti-inflammatory compounds of *A. rosea* flowers were clearly identified and validated. Compared with traditional chromatographic separation, the strategy of integrating UPLC/Q-TOF-MS/MS and bioactivity assay is more convenient and reliable. This strategy not only can be used for general component identification but also can directly screen trace active components from complex matrices.

## Materials and Methods

### Reagents and Chemicals

*Althaea rosea* flowers were purchased from Changan Chinese Herbal Medicine Co., Ltd. (Anguo, Hebei, China). The reporter plasmids pGL4.32 and pRL-TK were purchased from Promega (WI, United States). Human tumor necrosis factor alpha (TNF-α) was purchased from PeproTech (Rocky Hill, NJ, United States). Dexamethasone (Dex) was purchased from Sigma Chemical Co., Ltd. (St Louis, MO, United States). The ELISA kit (IL-6 and IL-8) was purchased from Biosource International Life Technologies Co., (Camarillo, CA, United States). All reagents for cell culture were purchased from Gibco Life Technologies (Rockville, MD, United States). The acetonitrile (CH_3_CN) for the UPLC/Q-TOF-MS/MS was UPLC-grade and was purchased from Thermo Fisher (Waltham, MA, United States). Deionized water was obtained by the Milli-Q system (Bedford, MA, United States). The reference compounds protocatechuic acid, caffeic acid, ferulic acid; quercetin, luteolin, naringenin, and kaempferol were purchased from Yifang Technologies (Tianjin, China). All other reagents used were of analytical grade.

### Sample Preparation

One hundred grams of *A. rosea* flowers was boiled in 1 L of 75% ethanol. The solution was filtered and evaporated to dryness (10.28 g). The concentrate was dissolved in water to a 10 mg/mL concentration and filtered through a filter (0.22 μm) membrane and stored at 4°C.

### Liquid Chromatography Conditions

The Waters Acquity UPLC Instrument System (Waters Co., United States) was controlled by MassLynx V4.1 software (Waters Co., United States). Separation was carried out using an Acquity BEH C_18_ column (2.1 mm × 100 mm, 1.7 μm; Waters Co., United States). The column temperature was maintained at 30°C, and the flow rate was 0.40 mL/min. The injection volume of the test sample was 2 μL. The mobile phase was CH_3_CN (A) and 0.5% aqueous formic acid (B), using a gradient elution. The steps were as follows: 2–11% A, 0–6 min; 11–16% A, 6–10 min; 16–17% A, 10–13 min; 17–19% A, 13–15 min; 19–25% A, 15–18 min; 25–100% A, 18–21 min; and 100% A in 21–22 min.

### Mass Spectrometry Analysis

The components of *A. rosea* flowers were identified using a Waters Q-TOF Premier and Electrospray Ionization (ESI) system (Waters MS Technologies, Manchester, United Kingdom). At the same time, ESI-MS spectra were obtained in positive ion and negative ion modes. The positive mode capillary voltage was set to 2.8 kV, and the negative mode was 2.2 kV. The sample cone voltage was set to 40 V, and the source temperature was 120°C. The sample atomization and auxiliary gas were high purity nitrogen. The rate of atomizing gas was 800 L/h, the cone gas was 50 L/h, and the degassing temperature was set to 400°C. The Q-TOF Premier acquisition rate was 0.1 s, and the inter-scan delay was 0.02 s. The argon gas used as the collision gas was maintained at a pressure of 5.3 × 10^-5^ Torr. The instrument was operated in wide-pass mode (100–1500 Da) using the first resolution quadrupole. Leucine enkephalin acetate was used as a reference material to lock the mass ([M+H]^+^ = 555.2931, [M-H]^-^ = 553.2775).

### Sample Preparation for Bioactivity Assay

The fractions separated by UPLC were collected into 96 deep well plates (2.2 mL) every 30 s and then evaporated to dryness at 40°C in a vacuum oven. The 44 dried residues were dissolved in Dulbecco’s Modified Eagle Medium (DMEM) cell culture medium (100 μL) for luciferase reporter activity assay. The collection process of the components was repeated five times for the activity test.

### Anti-inflammatory Activity Tests *in vitro*

HEK293T cells were purchased from the American Type Culture Collection (Rockville, MD, United States). The cells were cultured in DMEM containing 10% fetal calf serum (FBS), 0.1 mg/mL streptomycin and 100 U/mL penicillin, and grown at 37°C, 5% CO_2_. Plasmid pGL4.32 (100 ng per well) and Renilla luciferase reporter vector plasmid pRL-TK (9.6 ng per well) were co-transfected into HEK 293T cells using the NF-κB luciferase reporter plasmid pGL4.32 (100 ng per well).

The cells were incubated for 1 day before adding seven commercial anti-inflammatory compounds. The cells were pre-treated with the representative compounds (protocatechuic acid, caffeic acid, ferulic acid; quercetin, luteolin, naringenin, and kaempferol, 10 mg/L) and Dex (4 mg/L) for 1 h and then stimulated with TNF-α (10 ng/mL) for 6 h. After stimulating the cells, HEK 293T cells were lysed and assayed for luciferase activity using a dual luciferase reporter assay system according to the manufacturer’s instructions (Promega, Madison, WI, United States).

### Measurement of Chemokines (IL-6 and IL-8)

After stimulating the cells with TNF-α (10 μg/L), the potential bioactive compound (10 mg/L) and Dex (4 mg/L) were added, then commercial enzyme-linked immunosorbent assay kits (Pierce/Endogen, Rockford, IL, United States) were used to measure the release of IL-6 and IL-8 from the supernatants of HEK 293T cells. We collected the supernatant and measured the absorbance of each sample at 450 nm using a microplate reader (Bio-Rad Model 680, Hercules, CA, United States). The levels of chemokines (IL-6 and IL-8) were determined from the standard curve and expressed as pg/mL.

### Statistical Analysis

All assays were repeated three times and are presented as the mean ± standard deviation (SD) and analyzed by analysis of variance (ANOVA) followed by Dunnett’s test. The significance of the differences was determined using GraphPad Prism 5 (Graph Pad Software Inc., San Diego, CA, United States), and *p* < 0.05 was considered to be statistically significant.

## Results and Discussion

### Chemical Identification of *A. rosea* Flowers

**FIGURE 1:**
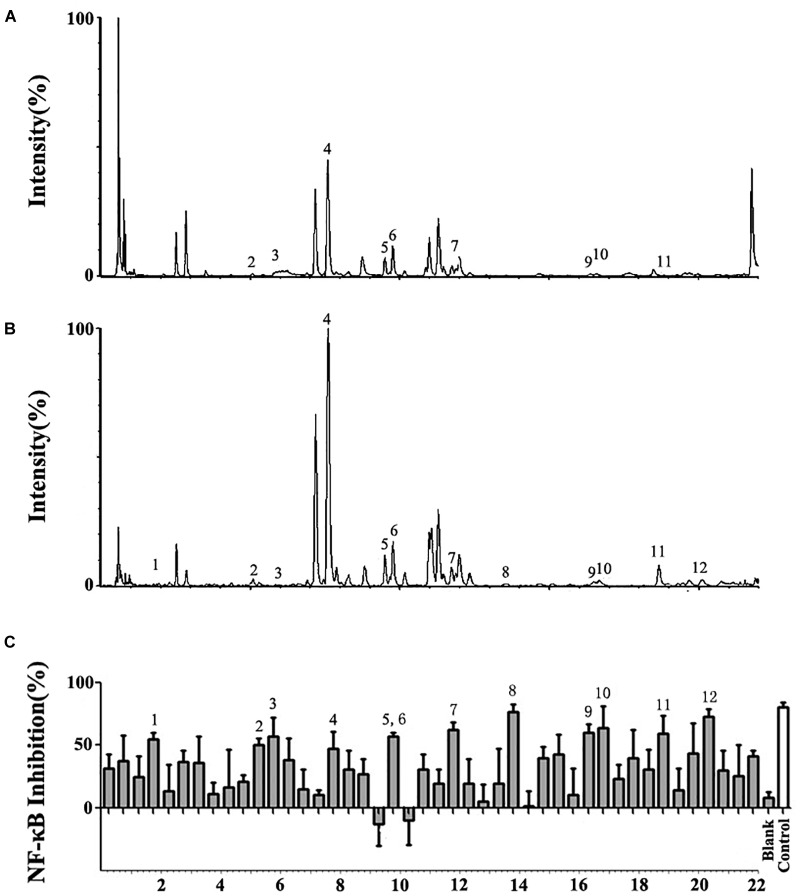
UPLC/Q-TOF-MS/MS and bioactivity analysis of the *Althaea rosea* flowers. **(A)** Total Ion Chromatography (TIC) chromatograms in positive ESI mode. **(B)** TIC chromatograms in negative ESI mode. **(C)** Bioactivity chromatograms obtained via the luciferase reporter assay system for NF-κB inhibition. The peak numbers are consistent with those reported in Table 1.

UPLC/Q-TOF-MS/MS was used to separate the extract of *A. rosea* flowers and obtain the accurate molecular weight of the components. Most of the chromatographic peaks were completely separated (Figures 1A,B). The mass spectra of the compounds were compared with the mass spectra of the standard or literature data to obtain 27 compounds (Supplementary Figure S1 and Supplementary Table S1 in the Supplementary information).

### Assay of NF-κB Inhibition

**FIGURE 2:**
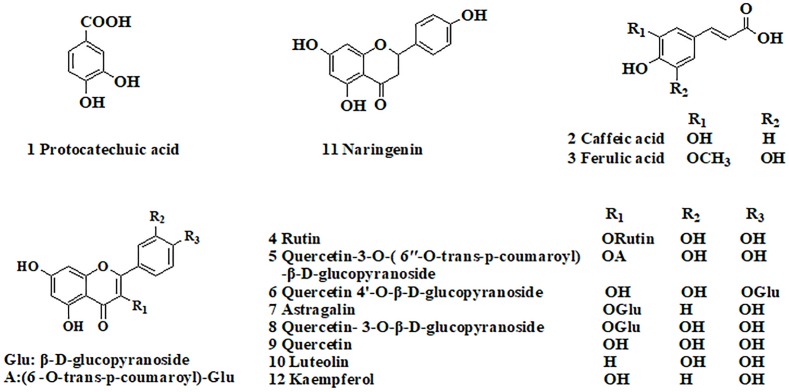
Chemical structures of the bioactive compounds in *Althaea rosea* flowers.

A total of 44 fractions were obtained by UPLC separation (Figure 1C). Through the activity test, some fractions caused significant inhibition of NF-κB. Detailed mass spectral information for these fractions is listed in Table 1, and 12 compounds were further identified based on their ionic fragmentation behavior. These compounds were divided into four groups according to their chemical structures: phenolic acid, hydroxycinnamic acid, flavonoid, and dihydroflavone (Figure 2). Potential anti-inflammatory compounds were quantified according to the labeling curve of the commercial standard, and the data are presented in Table 1. The highest content component was quercetin and its glycoside compound. The total content of quercetin and its derivatives reached 1.148%.

**Table 1:** MS/MS data in (±) ESI modes and the identification results for the bioactive compounds in Althaearosea.

	Time		MS-ESI	MS-ESI	Error				Content
Peak	(min)	Mode	(+) m/z	(-) m/z	(ppm)	MS/MS(m/z)	Composition	Component	(μg/g)
1	1.63	Neg		153.0505	2.072	153 [M-H]^-^	C_7_H_6_O_4_	Protocatechuic acid	113.7
						136 [M-H-H_2_O]^-^			
2	5.1	PN	181.0356	179.0353	3.314(+)	179[M-H]^-^	C_9_H_8_O_4_	Caffeic acid	340.9
						145[M-H-OH-OH]^-^			
						135[M-H-COO]^-^			
3	5.95	PN	195.0509	193.0505	4.144(+)	193[M-H]^-^	C_10_H_10_O_4_	Ferulic acid	295.6
						176[M-H-OH]^-^			
						149[M-H-OH-OCH_3_]^-^			
4	7.662	PN	611.1611	609.1606	0.654(+)	609[M-H]^-^	C_27_H_30_O_16_	Rutin	550.1
						463[M-H-Glu]^-^			
						301[M-H-Glu-]^-^			
5	9.5	PN	611.1388	609.1394	-1.145(+)	609[M-H]^-^	C_30_H_26_O_14_	Quercetin- 3- O- ( 6″- O- *trans*- p- coumaroyl) -β- D- glucopyranoside	4371
						301[M-H- coumaroyl-Glu]^-^			
6	9.51	PN	465.1031	463.1029	0.430(+)	463[M-H]^-^	C_21_H_20_O_12_	Quercetin 4′- O-β- D- glucopyranoside	3635
						927[2M-H]^-^			
						301[M-H-Glu]^-^			
7	11.89	PN	449.1001	447.1013	1.566(-)	895[2M-H]^-^	C_21_H_20_O_11_	Astragalin	79.14
						447[M-H]^-^			
						285[M-H-Glu]^-^			
8	13.56	Neg		463.1028	-0.432	463[M-H]^-^	C_21_H_20_O_12_	Quercetin- 3- O-β- D- glucopyranoside	2873
						301[M-H-Glu]^-^			
9	16.47	PN	303.0497	301.0506	2.325(+)	301[M-H]^-^	C_15_H_10_O_7_	Quercetin	606.1
						601[2M-H]^-^			
10	16.59	PN	287.0448	285.0456	-7.367(-)	285[M-H]^-^	C_15_H_10_O_6_	Luteolin	397.5
						331[M+HCOOH-H]^-^			
						571[2M-H]^-^			
11	18.96	PN	273.0694	271.0688	0.518	271[M-H]^-^	C_15_H_12_O_5_	Naringenin	285.2
						254[M-H-OH]^-^			
12	20.12	Neg		285.0463	4.911	285[M-H]^-^	C_15_H_10_O_6_	Kaempferol	87.13
						268[M-H-OH]^-^			
						571[2M-H]^-^			


*Neg, negative ion mode; PN, positive and negative ion mode.*

### Demonstration of the Bioactivity of NF-κB Inhibitors of *A. rosea* Flowers

**FIGURE 3:**
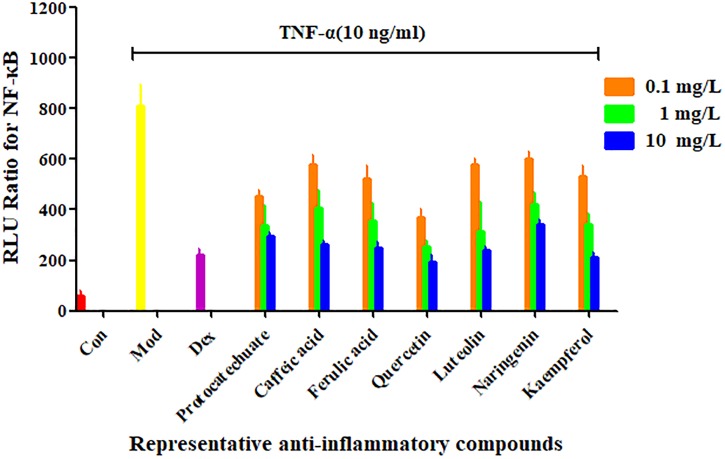
Confirmation of NF-κB inhibitors from *Althaea rosea* flowers by the luciferase reporter assay system. Each bar represents the mean ± SEM, *n* = 5 per group.

**FIGURE 4:**
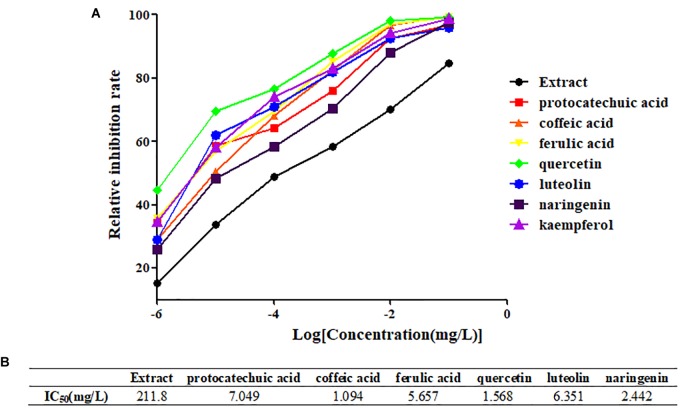
Determination of IC_50_ of representative compounds. **(A)** The inhibition curves of representative compounds. **(B)** The IC_50_ values of representative compounds.

From the four types of compounds, we selected seven compounds as representatives, namely, protocatechuic acid, caffeic acid, ferulic acid, quercetin, luteolin, naringenin, and kaempferol. A representative sample was added to TNF-α-stimulated HEK 293T and evaluated using a luciferase reporter assay system to investigate whether the compounds were effective in inhibiting the release of NF-κB. To evaluate inhibition of potential inhibitors in *A. rosea* flowers, Dex (4 mg/L) was used as a positive control. The inhibition profiles are shown in Figure 3. Compared with the model group, the seven compounds exhibited similar NF-κB inhibition (*p* < 0.05). As expected, the potential anti-inflammatory compounds were significantly more active than the extracts, with IC_50_ values of 7.049, 1.094, 5.657, 1.568, 6.351, and 2.442 mg/L, respectively. However, the extract had an IC_50_ value of 211.8 mg/L (Figure 4).

### Confirmation of Inhibition of Inflammatory Factor Production (IL-6 and IL-8)

**FIGURE 5:**
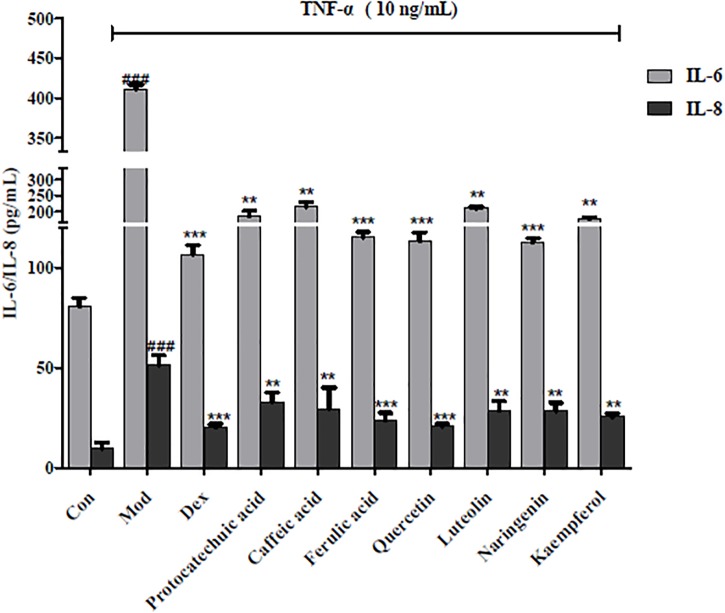
Effects of representative compounds of NF-κB inhibitor on IL-6 and IL-8 expression in TNF-α-induced HEK 293 T. Error bars indicate SEM, *n* = 4. ^∗∗^*p* < 0.01 vs. model group; ^∗∗∗^*p* < 0.001 vs. model group; ^###^*p* < 0.001 vs. control group.

Further measures of inflammatory factors (IL-6 and IL-8) were used to determine the anti-inflammatory effects of potential NF-κB inhibitors. As shown in Figure 5, all seven compounds reduced the expression of IL-6 and IL-8 (*p* < 0.05); further confirming the anti-inflammatory effects of the four structural types of compounds in *A. rosea* flowers. Among them, 10 μg/mL of ferulic acid, quercetin, and naringenin inhibited the production of inflammatory factors, which was very similar to the effect of 4 mg/L Dex. This indicates that ferulic acid, quercetin, and naringenin are relatively strong anti-inflammatory inhibitors in *A. rosea* flowers.

## Discussion

UPLC/Q-TOF-MS/MS has been developed and validated as an upgrade to traditional chromatographic methods for the separation and determination of trace substances in complex matrices (Wang et al., 2013). In this study, based on the combination of UPLC/Q-TOF-MS/MS technology and biological activity screening, we screened potential NF-κB inhibitors in *A. rosea* flowers rapidly and accurately. Subsequently, the inhibitory effect of potential inhibitors on inflammatory factor production was evaluated *in vitro*. Some of the compounds with high activity would likely be ignored when using traditional screening methods due to the low content when using the inhibition activity assay. Integrating MS identification and bioactivity validation was more sensitive and reliable than the conventional methods, not only in the detection and identification of constituents in a complex matrix but also in screening potential lead compounds. This method is sensitive, and the detailed method validation is complete. Information on potential inhibitors provided here will facilitate the development of new drugs and the development of plant resources (Qingxin et al., 2016). We demonstrate here that this integrated system has advantages in separation, structural identification, and bioactivity analysis of hit compounds (Halai and Cooper, 2012; Dong et al., 2013).

The 293T cell line is a derivative of the 293T (293tsA1609neo) cell line. 293T is a highly transfectable derivative of the 293 cell line into which the temperature sensitive gene for SV40 T-antigen was inserted. These cells constitutively express the simian virus 40 (SV40) large T antigen, and clone 17 was selected specifically for its high transfect ability (Hou et al., 2012). Therefore, we selected HEK293T as a vector for screening anti-inflammatory active compounds. NF-κB plays a key role in the expression of various pro-inflammatory genes caused by infections such as bacteria and viruses. The main consequence of inflammation is the upregulation of nuclear factor-κB (NF-κB) and subsequent damage. This upregulation leads to the synthesis of cytokines and chemokines, including interleukins IL-6, IL-8, RANTES, and IL-11, which in turn cause a series of stress responses to achieve inflammatory effects (Zyuz’kov et al., 2015). Although screening based on NF-κB inhibitory activity will help to identify effective and novel anti-inflammatory drugs, it is also likely to cause certain false positives (Cheng et al., 2012). Therefore, it is necessary to further confirm activity with the detection of downstream inflammatory factors such as IL-6 and IL-8.

We hypothesized that the 12 potential hit compounds are active ingredients, and the synergy between the various components leads to the specific anti-inflammatory effects of *A. rosea* flowers (Gao et al., 2015). Previous studies have shown that ferulic acid, caffeic acid, rutin, and quercetin can reduce the activation of NF-κB and have a significant anti-inflammatory effect (Cheng et al., 2012). In particular, quercetin exhibited a remarkable ability to inhibit the production of inflammatory factors such as NF-κB, IL-6, and IL-8 in this study. This strategy clearly demonstrates that mass spectrometry fingerprinting combined with bioactivity assays is a powerful tool for improving the screening and identification of potential active compounds in plants.

*Althaea rosea* is commonly used as a Uighur drug and ornamental plant with a small range of applications, and its cultivation range and economic value need to be further developed. It has been found that *A. rosea* flowers contain a strong anti-inflammatory compound, quercetin. The latest research demonstrates that quercetin has other important effects. Quercetin could selectively eliminate senescent cells, reduces the number of naturally occurring senescent cells, and secretes fragile-associated pro-inflammatory cytokines in human adipose tissue explants (Xu et al., 2018). Our study found that quercetin and its glycosides are concentrated in *A. rosea* flowers. This will certainly enhance the cultivation value of *A. rosea*, making it a botanical source of important health and pharmaceutical compounds.

## Conclusion

The high-efficiency separation and structural identification information provided by UPLC/Q-TOF-MS/MS, combined with the high sensitivity activity evaluation of the luciferase reporter gene detection system, enables simultaneous screening of trace samples obtained by chromatographic separation. The system has important application for the screening of trace active ingredients in complex systems. Using this integrated system, we screened and validated potential NF-κB inhibitors in *A. rosea* flowers. The anti-inflammatory effect and active ingredients of rose flower are clarified, which furthers our understanding of the medicine’s mechanism of action and promotes the development of lead compounds. In addition, the discovery of quercetin, an important compound in *A. rosea* flower, will also increase the planting value of *A. rosea*, which is conducive to the development of medicinal plants.

## Author Contributions

QC designed the study. FM performed the experiments, acquired and analyzed the data, and drafted and edited the manuscript. GB and QC contributed to data discussion and review of the manuscript.

## Conflict of Interest Statement

The authors declare that the research was conducted in the absence of any commercial or financial relationships that could be construed as a potential conflict of interest.
