# Supplementary material for: Combining UPLC/Q-TOF-MS/MS With Biological Evaluation for NF-κB Inhibitors in Uyghur Medicine Althaea rosea Flowers
Source: Front Plant Sci. 2019 Jan 9;9:1975. doi: 10.3389/fpls.2018.01975 (PMC6334161; doi:10.3389/fpls.2018.01975)
Supplement: Supplementary file 1 [file Data_Sheet_1.docx]

**Supplementary information**


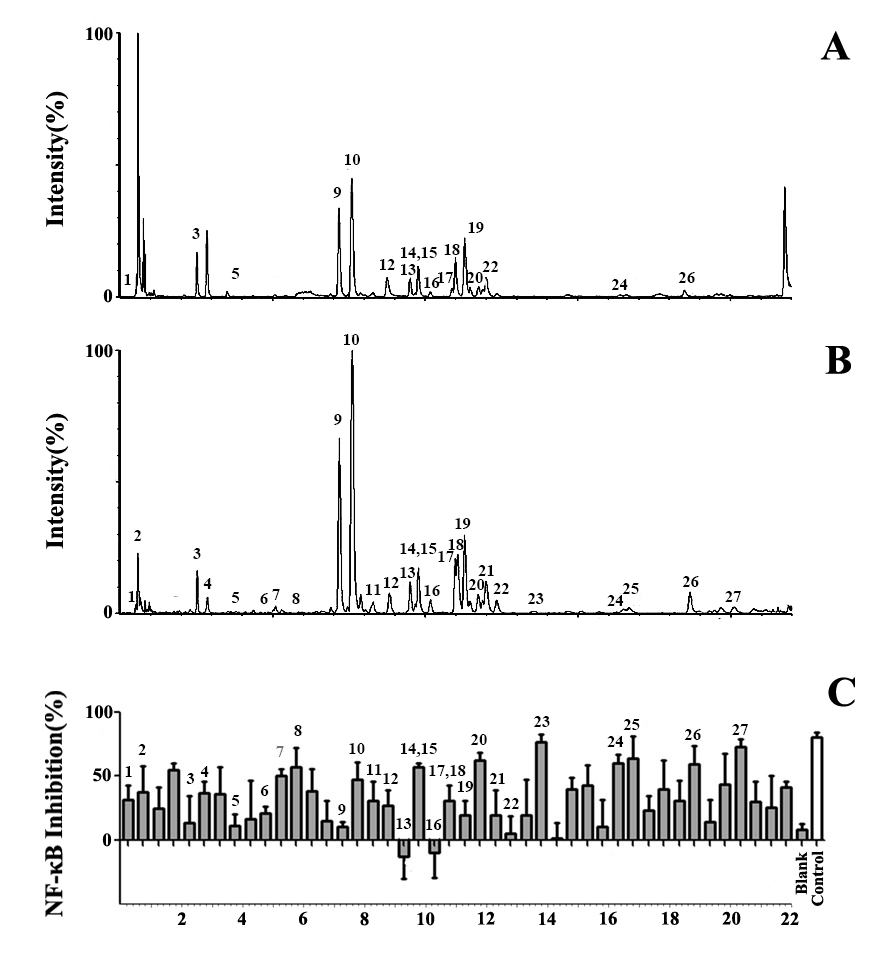


**Fig. S1**: UPLC/Q-TOF-MS and bioactivity analysis of the Althaea rosea flowers. (A) Total Ion Chromatography (TIC) chromatograms in positive ESI mode. (B) TIC chromatograms in negative ESI mode. (C) Bioactivity chromatograms obtained via the luciferase reporter assay system for NF-κB inhibition activation. The peak numbers are consistent with those reported in Table 1S.

**Table S1**. MS/MS data in (±) ESI modes and the identification results for the compounds in *Althaea rosea* flowers.

| Peak | Time | Mode | m/z | MS/MS(m/z) | Composition | Component |
| --- | --- | --- | --- | --- | --- | --- |
| 1 | 0.77 | PN | 152.1047 | 151 [M-H]^-^  134[M-H-OH]^-^ | C_8_H_8_O_3_ | p-hydroxyphenylacetic |
| 2 | 1.63 | Neg | 154.0266 | 153 [M-H]^-^  136 [M-H-H_2_O]^-^ | C_7_H_6_O_4_ | Protocatechuic acid |
| 3 | 2.27 | PN | 152.0473 | 107 [M-H-COO]^-^  134[M-H-OH]^-^ | C_8_H_8_O_3_ | anisic acid |
| 4 | 3.29 | Neg | 198.0528 | 197[M-H]^-^  153[ M-H-COO]^-^ | C_9_H_10_O_5_ | 3， 5- di methoxy- 4- hydroxy benzoic acid |
| 5 | 3.809 | PN | 138.0313 | 93[M-H-COO]^-^  137[M-H]^-^ | C_7_H_6_O_3_ | Salicylic acid |
| 6 | 4.857 | Neg | 418.1264 | 417[M-H]^-^  383[M-H-OH-OH]^-^ | C_21_H_22_O_9_ | isoliquiritin |
| 7 | 5.1 | Neg | 180.0423 | 179[M-H]^-^  145[M-H-OH-OH]^-^  135[M-H-COO]^-^ | C_9_H_8_O_4_ | Caffeic acid |
| 8 | 5.95 | Neg | 194.0579 | 193[M-H]^-^  176[M-H-OH]^-^  149[M-H-OH-OCH_3_]^-^ | C_10_H_10_O_4_ | Ferulic acid |
| 9 | 7.18 | PN | 466.1111 | 465[M-H]^-^  303[M-H-Glu-]^-^ | C_21_H_22_O_12_ | (2R,3R)-taxifolin-7-O-β-D-glucopyranoside |
| 10 | 7.662 | PN | 610. 1534 | 609[M-H]^-^  463[M-H-Glu]^-^  301[M-H-Glu-]^-^ | C_27_H_30_O_16_ | Rutinum |
| 11 | 8.03 | Neg | 480.0904 | 479[M-H]^-^  317[M-H-Glu-]^-^ | C_21_H_20_O_13_ | myricetin- 3- O-β- D- glucopyranoside |
| 12 | 8.23 | PN | 466.111 | 465[M-H]^-^  303[M-H-Glu-]^-^ | C_21_H_22_O_12_ | (2R,3R)-taxifolin-3′-O-  β-D-glucopyranoside |
| 13 | 8.821 | PN | 304.0583 | 303[M-H]^-^  287[M-OH]^-^ | C_15_H_12_O_7_ | (2R,3R)-(+)-taxifolin |
| 14 | 9.5 | PN | 610.1323 | 609[M-H]^-^  301[M-H-coumaroyl-Glu] ^-^ | C_30_H_26_O_14_ | Quercetin- 3- O- ( 6″- O- trans- p- coumaroyl) -β- D- glucopyranoside |
| 15 | 9.51 | PN | 464.0955 | 463[M-H]^-^  927[2M-H]^-^  301[M-H-Glu]^-^ | C_21_H_20_O_12_ | Quercetin 4'- O-β- D- glucopyranoside |
| 16 | 10.23 | PN | 594.1585 | 593[M-H]^-^  285[M-H-rutinoside]^-^ | C_27_H_30_O_15_ | Kaempferol 3-O-rutinoside |
| 17 | 10.98 | PN | 594.1373 | 593[M-H]^-^  285[M-H- coumaroyl-Glu]^-^ | C_30_H_26_O_13_ | kaempferol-3-O-(6 –O-  trans-p-coumaroyl)-β-D-glucopyranoside |
| 18 | 11 | PN | 594.1449 | 593[M-H]^-^  285[M-H- coumaroyl-Glu]^-^ | C_30_H_26_O_13_ | kaempferol-3-β-O-(6’’ –O-cis-p-coumaroy) -D-glucopyranoside |
| 19 | 11.29 | PN | 448.1006 | 447[M-H]^-^  285[M-H-Glu]^-^ | C_21_H_20_O_11_ | luteolin-4’-O-β-D-glucopyranoside |
| 20 | 11.89 | PN | 448.1006 | 895[2M-H]^-^  447[M-H]^-^  285[M-H-Glu]^-^ | C_21_H_20_O_11_ | Astragalin |
| 21 | 12.00 | PN | 432.1056 | 431[M-H]^-^  269[M-H-Glu]^-^ | C_21_H_20_O_10_ | apigenin-4'- O-β- D- glu copyranoside |
| 22 | 12.37 | PN | 448.1006 | 447[M-H]^-^  269[M-H-Glu]^-^ | C_21_H_20_O_11_ | Kaempferol-4’-O- -D-glucopyranoside |
| 23 | 13.56 | Neg | 464.0955 | 463[M-H]^-^  301[M-H-Glu]^-^ | C_21_H_20_O_12_ | Quercetin- 3- O-β- D- glucopyranoside |
| 24 | 16.47 | PN | 302.0427 | 301[M-H]^-^  601[2M-H]^-^ | C_15_H_10_O_7_ | Quercetin |
| 25 | 16.59 | Neg | 286.0477 | 285[M-H]^-^  331[M+HCOOH-H]^-^  571[2M-H]^-^ | C_15_H_10_O_6_ | Luteolin |
| 26 | 18.96 | PN | 272.0685 | 271[M-H]-  254[M-H-OH]^-^ | C_15_H_12_O_5_ | Naringenin |
| 27 | 20.12 | Neg | 286.0447 | 285[M-H]^-^  268[M-H-OH]^-^  571[2M-H]^-^ | C_15_H_10_O_6_ | Kaempferol |

Neg: Negative ion mode, PN: Positive and negative ion mode
